# Quality of Life After Pancreatic Surgery for Neuroendocrine Tumors of the Pancreas: Observational Study of Long-Term Outcomes

**DOI:** 10.3390/cancers17193205

**Published:** 2025-10-01

**Authors:** Anna Caterina Milanetto, Claudia Armellin, Daniele Gasparini, Giulia Lorenzoni, Claudio Pasquali

**Affiliations:** 1 Pancreatic and Digestive Endocrine Surgery, Department of Surgery, Oncology and Gastroenterology, University of Padua, Via Giustiniani 2, 35128 Padua, Italy; claudio.pasquali@unipd.it; 2 Department of Medicine, UniCamillus International Medical University in Rome, Via di Sant’Alessandro 8, 00131 Rome, Italy; 3 Department of General Surgery, Policlinico di Abano Terme, Piazza Cristoforo Colombo 1, 35031 Abano Terme, Italy; carmellin@casacura.it; 4 Unit of Biostatistics, Epidemiology and Public Health, Department of Cardiac, Thoracic, Vascular Sciences and Public Health, University of Padova, Via Loredan 18, 35131 Padua, Italy; daniele.gasparini@ubep.unipd.it (D.G.); giulia.lorenzoni@unipd.it (G.L.)

**Keywords:** pancreas, neuroendocrine tumor, surgery, quality of life

## Abstract

**Simple Summary:**

One hundred patients operated on for a pancreatic neuroendocrine tumor (1990–2023) received three EORTC questionnaires (QLQ-C30 and the new P.NET15 and P.NET19) to evaluate quality of life outcomes after surgery in relationship with clinical variables. After a median time of 133 months after surgery, patients showed a good QoL (median 83.3/100). Elderly and diabetic patients who underwent a standard pancreatic resection for a gastrinoma/non-functioning neuroendocrine tumor showed a significant worsening of QoL outcomes.

**Abstract:**

**Background/Objectives**: Patients with pancreatic neuroendocrine tumors (PanNETs) often have a good prognosis with long overall survival. We evaluated quality of life (QoL) after surgery for PanNETs, using the new EORTC-specific questionnaires. **Methods**: PanNET patients operated on in our unit (1990–2023) received three EORTC questionnaires (QLQ-C30 and the new P.NET15 and P.NET19). We evaluated the following: (1) QLQ-C30 outcomes; (2) mixed domains from QLQ-C30, P.NET15, and P.NET19; and (3) domains from P.NET19 and P.NET15 only. Functional and symptom scales were investigated in relationship with clinical variables. Gamma regression and multivariable analyses were performed with R software. **Results**: The 100 patients enrolled (median time 133 months after surgery) showed a good QoL (median 83.3/100). Old age was related to worse QoL and physical functioning (*p* = 0.007 and *p* < 0.001, respectively). Diabetes negatively influenced QoL (*p* < 0.001), physical functioning (*p* = 0.005), and fatigue (*p* = 0.03). Patients undergoing parenchyma-sparing surgery showed less fatigue (*p* = 0.046), while non-insulinoma PanNET diagnosis was related to worse QoL (*p* = 0.039). Multiple comorbidities were negatively associated with physical functioning (*p* = 0.010), fatigue (*p* = 0.001), and pain (*p* = 0.021). According to the new questionnaires, the most affected outcome was muscle energy, depending on age (*p* = 0.042), diabetes (*p* = 0.014), type of surgery (*p* = 0.018), and non-insulinoma diagnosis (*p* = 0.007). **Conclusions**: A good QoL evaluated with EORTC questionnaires is reported in PanNET patients after surgery. Elderly and diabetic patients who underwent standard resection for gastrinoma/non-functioning PanNETs showed worse QoL outcomes.

## 1. Introduction

Pancreatic neuroendocrine tumors (PanNETs) account for < 2% of all pancreatic tumors, but their incidence is increasing. They may be associated with a clinical syndrome (i.e., insulinoma and gastrinoma) or non-functioning tumors (NF-PanNETs), which represent the vast majority (60–70%) [1]. More than 15% of them are part of an inherited syndrome, mostly multiple endocrine neoplasia type 1 (MEN1) [2]. The most important prognostic factors are TNM stage and tumor grade [3], the latter based on proliferative index according to the WHO classification system [4,5].

Surgery is the only curative option among all the available therapeutic approaches (i.e., somatostatin analogs, targeted therapies, peptide receptor radionuclide therapy, chemotherapy, and liver-directed treatments), which are instead preferred for high-grade PanNETs, in a metastatic setting, or in patients unfit for surgery [1,3,6]. An accurate patient selection and a tailored surgical approach are crucial to achieve the best oncological outcomes so that patients may show a favorable course after surgery, even in the presence of liver metastases or grade 2–3 PanNETs [3].

Health-related quality of life (HRQoL) is a patient-reported outcome that should be considered along with survival outcomes, especially in PanNET patients, due to their better prognosis when compared to other cancer patients. Some previous studies reported on HRQoL in patients affected by gastro–entero-pancreatic (GEP) NETs, and PanNETs were evaluated together with gastric and intestinal NETs [7,8,9,10,11], except for insulinomas [12]. The European Organization for Research and Treatment of Cancer (EORTC) is a leading reference in evaluating HRQoL of patients in neoplastic settings, which developed specific modules for different cancer populations [13].

In a previous study we investigated HRQoL in a similar series of PanNET patients after surgery, using EORTC QLQ-C30, GI.NET21, and PAN26 questionnaires [14]. In spring 2023, EORTC finally released two specific questionnaires regarding PanNETs: EORTC QLQ-P.NET19 Insulinoma (P.NET19) and EORTC QLQ-P.NET15 Gastrinoma/Non-Functioning (P.NET15), which are still in the validation phase [15]. As generic questionnaires may overlook peculiar issues of PanNET patients, we decided to perform a similar investigation to before using the new specific questionnaires.

The aim of this observational study was to investigate HRQoL determinants in patients surgically treated for a PanNET and how different clinical features related to patients, disease, and treatments may affect the HRQoL outcomes. Finally, we compared the different pools of questions (called Analyses 1, 2, and 3), combining items from the QLQ-C30, P.NET15, and P.NET19 questionnaires, to evaluate which of them may highlight significant results and may be the most appropriate to assess HRQoL in this PanNET population.

## 2. Materials and Methods

### 2.1. Study Population

Patients who underwent surgery for a PanNET between 1 January 1990 and 30 June 2023 in our Pancreatic Surgical Unit were identified from hospital computerized files. Inclusion criteria were a histologically confirmed diagnosis of PanNET, age between 18 and 85 years, and at least six months of follow-up (FU) after surgery. Clinical records of the included patients were retrieved: (1) at the time of surgery: gender, MEN1 diagnosis, tumor functional state, tumor grade, and type of surgery; (2) at the time of the questionnaires: age, other diseases, tumor burden, other treatments, and pancreatic endocrine and exocrine function. Most patients (71%) were enrolled during the outpatient clinic FU visit, while the others were contacted by phone and sent the questionnaires by mail. This study was approved by the local Ethical Committee (reference number 5091/AO/21), and written informed consent was obtained from all the patients. Enrollment of patients, questionnaire administration, and clinical data collection were carried out until 30 June 2024. This study was designed to check the adherence to the STROBE statement [16].

### 2.2. Health-Related Quality of Life Assessment and Rationale

To assess HRQoL in PanNET patients, we used the Italian version of the EORTC QLQ-C30, P.NET19, and P.NET15 questionnaires from the EORTC website. The EORTC QLQ-C30 is a general QoL questionnaire composed of 30 items for all cancer patients, grouped into functional and symptom scales. The P.NET19 is composed of 19 items, while the P.NET15 is composed of 15 items; all these items were considered as belonging to symptom scales. They share the same first 10 items common to PanNET patients, while the other nine and five items are specific for insulinoma and gastrinoma/NF-PanNET (Gas/NF) patients, respectively. The scores for each item of the three questionnaires were transformed into a 0–100 linear scale according to the EORTC QLQ Scoring Manual [17]. For the functional scales and global QoL, a high score represents a high level of functioning (i.e., better HRQoL), whereas for the symptom scales, a high score indicates a high level of symptoms (i.e., worse HRQoL).

Clinical variables were considered binary whenever possible, as follows: gender (female vs. male), PanNET type (non-functioning vs. functioning), tumor grade (G2 vs. G1), type of surgery (standard vs. parenchyma-sparing resection), other treatments after surgery (somatostatin analogs, systemic/locoregional treatment, and/or redo surgery: yes vs. no), and presence of active disease (combining patients with MEN1 diagnosis, local recurrence, and/or distant metastasis: yes vs. no). Patients with MEN1 diagnosis (and concomitant hyperparathyroidism, pituitary disease, etc.) and patients with local and/or distant tumor recurrence have been combined together in the same “active disease” category to improve statistical analysis quality due to the low number of cases of each sub-category, and also because having a disease included in MEN1 syndrome may influence HRQoL as much as having active oncologic disease.

Comorbidities were categorized as no vs. single vs. multiple, while postoperative pancreatic function was considered either as normal vs. exocrine/endocrine insufficiency together, or normal vs. exocrine insufficiency vs. diabetes mellitus. Age at questionnaire (in years) and time after surgery (in months) were considered as continuous variables. Other treatments and active disease carried similar information; therefore, they were considered as a single clinical variable for the subsequent analyses.

### 2.3. Domains’ Creation and Three Analyses

Statistical analyses were carried out after data conversion, grouping the single items describing the same topic into global domains. The EORTC QLQ-C30 questionnaire’s items were grouped according to the EORTC QLQ-C30 Reference Values Manual [18]. For the two new questionnaires, the EORTC QoL Academic Requests Section was reached via mail in July 2024, and as suggested, domain creation was adapted to the only existing literature reference by Ramage et al. [15]. The final score for each domain of the three questionnaires was calculated as the weighted average of the items’ scores included in that domain, and missing data were managed according to the EORTC QLQ-C30 Scoring Manual [17].

Three different analytic strategies were designed to evaluate which of the questionnaires could be the most appropriate to assess HRQoL in this PanNET population. First, in the primary analysis (called “Analysis 1”), QLQ-C30 data were considered separately, in order to obtain a basal evaluation of HRQoL in PanNET patients. Then, we created a new 40-item PanNET questionnaire, including the most common issues in both the general neoplastic population (30 items from QLQ-C30) and PanNET patients (the first 10 items of P.NET19 and P.NET15). This constructed mixed 40-item PanNET questionnaire was set up with an explorative purpose (therefore without any previous validation) to provide the patients a single tool to possibly increase their compliance in filling out the HRQoL evaluation (exploratory analysis, called “Analysis 2”). Finally, a focused and supportive analysis (called “Analysis 3”) was carried out; only the two new questionnaires were considered, grouping the items according to the existing literature [15].

**Figure 1:**
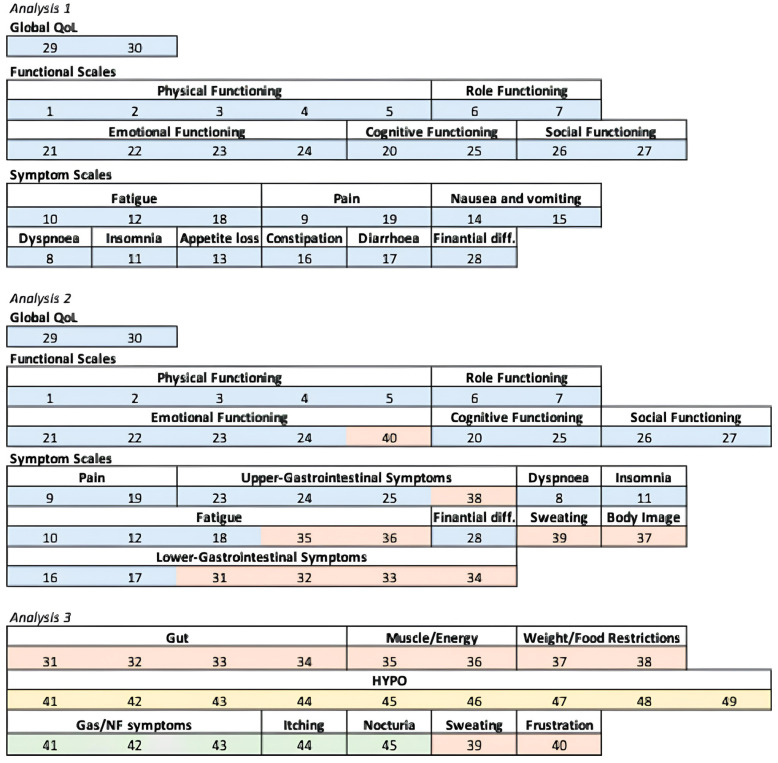
Domain elaboration used for Analyses 1, 2, and 3. The single items of each questionnaire are colored differently: blue boxes are EORTC QLQ-C30 items, orange boxes are the first 10 items of both EORTC P.NET19 and EORTC P.NET15, yellow boxes are the last nine items of EORTC P.NET19, and green boxes are the last five items of EORTC P.NET15. Global QoL, global quality of life. HYPO, low blood glucose/neurological symptoms. Gas/NF symptoms, non-functioning pancreatic neuroendocrine tumor, and gastrinoma-related symptoms.

Regarding P.NET19 and P.NET15, we grouped in a single domain (called “Specific symptoms”) the following domains: HYPO (low blood glucose/neurological symptoms), Gas/NF symptoms (NF-PanNET and gastrinoma-related symptoms), Itching, and Nocturia, in order to include the whole cohort of 100 patients for Analysis 3. The domain elaboration used for Analyses 1, 2, and 3 is shown in Figure 1.

For Analyses 1 and 2, among all the domains, we selected those to be considered for the subsequent statistical analyses, according to the current literature and to their relevance in the field of PanNETs. Primary endpoints of Analysis 1 were global QoL, physical functioning (PF2), emotional functioning (EF), pain, and fatigue. Primary endpoints of Analysis 2 were global QoL, PF2, EF, upper gastrointestinal (GI) symptoms, and lower GI symptoms. The selected domains were evaluated as outcome variables in relation to the clinical predictor variables. For Analysis 3 we investigated all the domains to provide complete data that may help in the validation of the new questionnaires P.NET19 and P.NET15. Therefore, primary endpoints of Analysis 3 were gut, muscle/energy, weight and food restrictions, sweating, frustration, HYPO, Gas/NF symptoms, itching, nocturia, and specific symptoms.

Similarly, in the univariable and multivariable analyses, we included only those predictor variables considered clinically relevant for each analysis. Multivariable analysis included the same outcome variables considered for univariable analysis for both Analysis 1 and Analysis 2, whereas for Analysis 3, the selected outcomes were Gut, Muscle/energy, and Specific symptoms.

### 2.4. Statistical Analysis

Descriptive statistics were reported as median (I–III quartiles) for continuous variables and percentages (absolute numbers) for categorical variables. The association between baseline characteristics and HRQoL scores (univariable analysis) was evaluated using the univariable Gamma model to account for the non-normal distribution of the outcomes, using a constant offset to overcome and handle zero values. The marginal effect was computed considering the partial derivatives of the marginal expectation. Results were reported as average marginal effect (AME), 95% CI, and *p*-value.

The AME represents the mean change in the outcome variables (functional or symptom scales) per unit increase in the independent variable. Positive values of AME have an improving meaning in global QoL and functional scales but a detrimental meaning in symptom scales, whereas negative values of AME have a detrimental meaning in global QoL and functional scales but an improving meaning in symptom scales. Results were also reported as minimal clinically important differences (MCIDs) for EORTC QLQ-C30. The MCID thresholds were chosen with an anchor-based “legacy” approach, according to Osoba et al. [19], who reported on the significance to patients of changes in HRQoL scores assessed by the EORTC QLQ-C30, with the cutoffs 5 (“a little”), 10 (“moderate”), and 20 (“very much”). False-discovery rate (FRD, Benjamini–Hochberg) adjustment, for multiplicity control, was applied within the outcome.

In the multivariable analysis, some outcome variables (different for each analysis) were evaluated in relation to a set of predictor variables (different for each analysis), selected in relation to the clinical relevance and complying with a minimum number of 10 patients (observations) per degree of freedom of the multivariable model, or 14−16 patients per predictor [20].

To validate the use of the Gamma model in this analysis, with large mass at zero data, a sensitivity analysis was performed, in which each HRQoL scale was analyzed using models appropriate for bounded outcomes with a mass at zero. For outcomes expressed on 0–100 scales (according to the EORTC QLQ-C30 manual), the following were fitted: (1) The main model, Gamma GLMs with log link to estimate effects on the mean of strictly positive responses, using a constant offset (after a sensitivity analysis, the chosen approach to produce results more concordant with the other models and avoid biased effect sizes was to substitute 0 with 1, on a 0−100 scale); (2) a two-part (hurdle) specification, on a 0−100 scale, that first models the probability of a structural zero (using a penalized likelihood method) and then the conditional mean among positives, with the marginal effect constructed on the overall (unconditional) mean [21]. For outcomes expressed on a 0–1 scale, the following were fitted: (1) Beta regression for proportions in (0, 1), with Smithson–Verkuilen boundary adjustment for 0/1 [22], and (2) fractional logit models for proportions including boundary values 0 or 1 [23]. Average marginal effects (AMEs) were reported on the response scale and, for comparability across models, were rescaled to “points” on the original 0–100 metric where applicable, for the Beta and fractional logistic models.

Uncertainty was quantified with model-based standard errors; for the two-part model, we were allowed either normal-approximation *p*-values or non-parametric bootstrap *p*-values (empirical, percentile CIs), and it was prespecified which *p*-value was used for multiplicity control, opting for the normal-approximation *p*-value because the other choices were too conservative. False-discovery rate (Benjamini–Hochberg) adjustment was applied within the outcome. Multiple sensitivity checks were performed (not reported): (1) rescaling conventions (fitting Beta/FracLogit on 0–1 while reporting AMEs back on 0–100); (2) alternative small offsets for zeros when required by specific estimators (Gamma model); (3) normal-approximation vs. bootstrap *p*-value comparison in the two-part model. Concordance across modelling families was summarized via correlation of AMEs, sign agreement, the Jaccard index of “significant” sets, and Cohen’s κ on significance flags, plus the Pearson correlation of signed Z-scores and the Spearman correlation of *p*-values.

Internal consistency and structural validity (dimensionality) were evaluated separately for insulinoma (P.NET-19) and gastrinoma/non-functioning (P.NET-15) symptom items. A parallel analysis was used to determine the number of factors, followed by exploratory factor analysis, and Cronbach’s α was reported, with corrected item–total correlations. As a secondary check, these analyses were repeated on a common symptom core (gut, muscle/energy, weight/food restriction, sweating, frustration) and on the common core plus a composite “Specific symptoms” score (defined as the mean of disease-specific items within P.NET-15 or P.NET-19; common items were not used to form the composite) [24,25,26]. Analyses were performed with R software v 4.4.0 together with the package margins [27].

## 3. Results

### 3.1. Clinical Data

**Figure 2:**
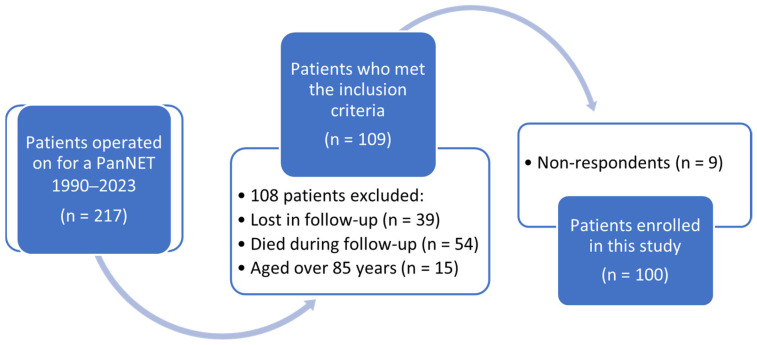
Flow diagram illustrating the patients’ selection process. PanNET, pancreatic neuroendocrine tumor.

Between 1990 and 2023, 217 patients were operated on for a PanNET in our Pancreatic Surgical Unit, and finally, among 109 patients who met the inclusion criteria and received the questionnaires, 100 patients provided all the questionnaires and were enrolled in this study (response rate 91.7%). There were no personal, clinical, or treatment differences between the respondents and non-respondents, but all the nine patients who did not return the questionnaires had received them by email (Figure 2 and Table 1).

### 3.2. Analysis 1

**Figure 3:**
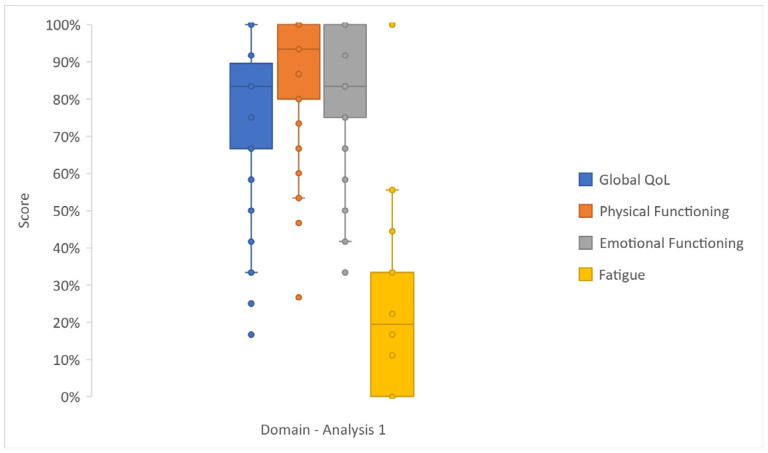
Box and whisker plot reporting the scores—as median, lower (Q1) and upper (Q2) quartiles, and minimum and maximum values—of selected domains of Analysis 1 (EORTC QLQ-C30 data).

Patients showed good global QoL results (median value 83%) in both Analyses 1 and 2. In Analysis 1 (Figure 3 and Table S1), PF2 (median 93%, IQR 80–100%) and EF (median 83%, IQR 75–100%) were modestly affected, and symptom scales generally showed low scores.

According to Gamma regression in Analysis 1 (Table S2), old age at FU negatively affected both global QoL and PF2 (AME −0.38 [−0.66; −0.10], *p*-value 0.007; and AME −0.45 [−0.67; −0.23], *p*-value < 0.001, respectively), as well as other diseases at FU negatively affected PF2 (AME −14.13 [−24.84; −3.41], *p*-value 0.010), pain (AME 8.28 [1.27; 15.29], *p*-value 0.021), and fatigue (AME 11.77 [4.81; 18.73], *p*-value 0.001). Diabetes diagnosis at FU considerably affected HRQoL outcomes, with a worsening of global QoL (AME −15.15 [−23.63; −6.68], *p*-value < 0.001), PF2 (AME −10.36 [−17.55; −3.17], *p*-value 0.005), and fatigue (AME 10.45 [1.00; 19.91], *p*-value 0.030), while exocrine insufficiency at FU was positively associated with fatigue (AME −8.41 [−15.31; −1.53], *p*-value 0.016). Standard surgical resections negatively affected fatigue (AME 7.10 [0.11; 14.11], *p*-value 0.046), while Gas/NF diagnosis negatively affected both global QoL (AME −9.67 [−18.82; −0.51], *p*-value 0.039) and fatigue (AME 8.15 [1.51; 14.80], *p*-value 0.016). Time from surgery to questionnaires did not affect QoL outcomes. All the above-mentioned statistically significant results represented “a little” to “moderate” changes compared to the related MCIDs (Table S2).

In the multivariable analysis (Figure S1), six clinical variables (active disease at FU, age at FU, gender, other diseases at FU, pancreatic function at FU, and time from surgery) were evaluated in relation to five outcomes (global QoL, PF2, EF, fatigue, and pain). Diabetes diagnosis at FU was confirmed as negatively correlating with global QoL (AME −12.17 [−21.70; −2.63], *p*-value 0.012), as PF2 was negatively influenced by older age at FU (AME −0.42 [−0.74; −0.11], *p*-value 0.008). Fatigue was negatively affected by multiple comorbidities at FU (AME 9.43 [0.38; 18.47]), while fatigue and pain were positively affected by exocrine insufficiency at FU (AME −10.35 [−16.73; −3.96], *p*-value 0.001, and AME −7.06 [−13.39; −0.73], *p*-value 0.029, respectively).

### 3.3. Analysis 2

**Figure 4:**
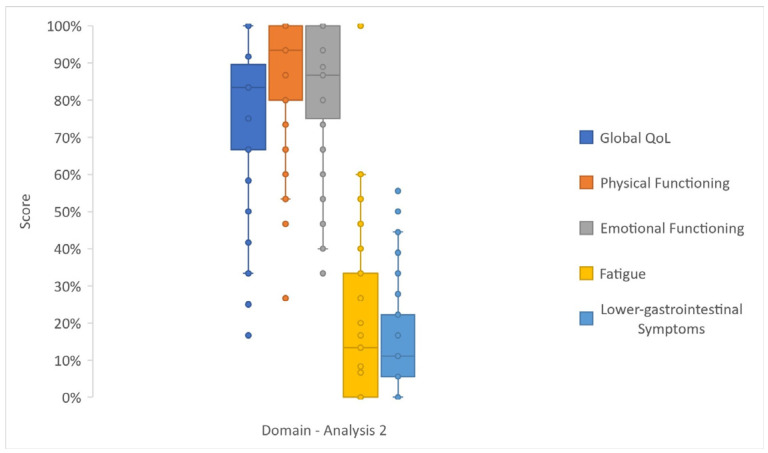
Box and whisker plot reporting the scores—as median, lower (Q1) and upper (Q2) quartiles, and minimum and maximum values—of selected domains of Analysis 2 (data coming from all items of EORTC QLQ-C30 and the first 10 items of EORTC P.NET19 and EORTC P.NET15).

Analysis 2 reported similar results to Analysis 1, with good functional scores and limited GI symptoms and fatigue (Figure 4 and Table S1).

Gamma regression in Analysis 2 (Table S3) showed a negative effect of old age at FU on PF2 (AME −0.45 [−0.67; −0.23], *p*-value < 0.001) and global QoL (AME −0.38 [−0.66; −0.10], *p*-value 0.006). Global QoL was worsened also by Gas/NF diagnosis (AME −9.67 [−18.82; −0.51], *p*-value 0.038) and by diabetes diagnosis at FU (AME −15.15 [−23.63; −6.68], *p*-value < 0.001), while PF2 was also worsened by other diseases at FU (AME −14.13 [−24.84; −3.41], *p*-value 0.009) and diabetes diagnosis at FU (AME −10.36 [−17.55; −3.17], *p*-value 0.004). Time from surgery to questionnaires did not affect QoL outcomes.

The multivariable analysis (Figure S2) showed no significant correlation between the selected clinical variables (gender, other diseases at FU, type of NET, and time from surgery) and EF or upper GI symptoms. Global QoL was worsened by diabetes diagnosis at FU (AME −10.41 [−20.28; −0.54], *p*-value 0.039), PF2 was worsened by old age at FU (AME −0.42 [−0.74; −0.11], *p*-value 0.009), and lower GI symptoms were positively affected by the presence of active disease at FU (AME −7.76 [−14.44; −1.08], *p*-value 0.023).

### 3.4. Analysis 3

**Figure 5:**
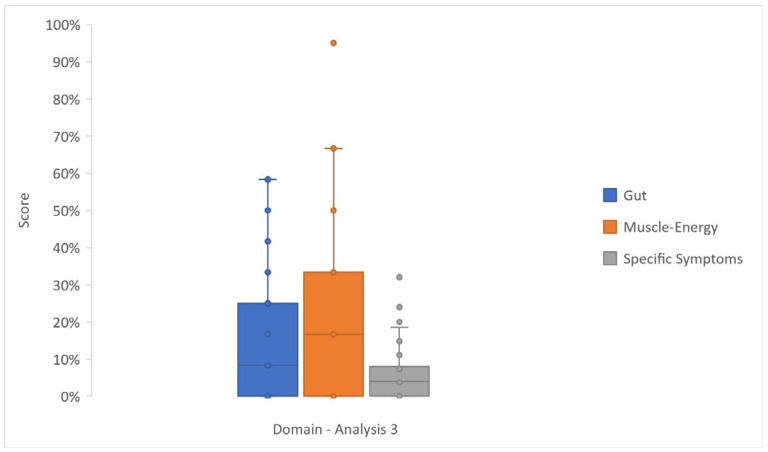
Box and whisker plot reporting the scores—as median, lower (Q1) and upper (Q2) quartiles, and minimum and maximum values—of selected domains of Analysis 3 (EORTC P.NET19 and EORTC P.NET15 data).

In Analysis 3 (Figure 5 and Table S4), the first 10 items of questionnaires P.NET19 and P.NET15 involved 100 patients, who showed a low incidence of symptoms. The insulinoma subgroup (n = 32) answered the last nine items of the P.NET19, providing a low score of HYPO (median 0%, IQR 0–4%), whereas the Gas/NF subgroup (n = 68) responded to the P.NET15 last five items, rarely complaining of symptoms (median 4%, IQR 0–8%).

Gamma regression for Analysis 3 (Table S5) highlighted the worsening effect of old age at FU on muscular/general energy (AME 0.32 [0.01; 0.63], *p*-value 0.042). The diagnosis of Gas/NF negatively influenced muscle/energy (AME 10.39 [2.82; 17.96], *p*-value 0.007) and also specific symptoms (AME 2.39 [0.14; 4.64], *p*-value 0.037). While diabetes diagnosis at FU negatively correlated with muscle/energy (AME 15.88 [3.21; 28.54], *p*-value 0.014), exocrine insufficiency at FU was related to better scores in muscle/energy (AME −9.31 [−15.89; −2.71], *p*-value 0.005). Standard pancreatic resections negatively affected muscle/energy (AME 10.40 [1.73; 19.06], *p*-value 0.018).

In multivariable analysis (Figure S3), three outcomes (gut, muscle/energy, and specific symptoms) were correlated with six clinical variables (active disease at FU, age at FU, pancreatic function at FU, type of surgery, type of NET, and time from surgery). Gas/NF diagnosis negatively affected specific symptoms (AME 2.56 [0.19; 4.92], *p*-value 0.034) and gut (AME 7.11 [0.46; 13.77], *p*-value 0.036). Gut was positively affected by active disease and age (AME −10.02 [−18.26; −1.78], *p*-value 0.017, and AME −0.36 [−0.68; −0.04], *p*-value 0.027, respectively). Muscle/energy was negatively influenced by diabetes at FU (AME 22.53 [5.64; 39.42], *p*-value 0.009). Time from surgery negatively affected the gut (AME 0.05 [0.01; 0.10], *p*-value 0.016) but was related to better scores in muscle/energy (AME −0.07 [−0.13; −0.01], *p*-value 0.044).

### 3.5. Sensitivity Analysis for Gamma Model

We harmonized scales by fitting Beta [22] and fractional logit [23] models on 0–1 as required and rescaling marginal effects to the 0–100 QoL metric; Gamma and two-part models were fit and reported on 0–100. Across 674 overlapping predictor–outcome contrasts, between-model correlations of AMEs were very high (r = 0.93–1.00), with 92–99.7% sign agreement and with the two-part model differing more than others, reflecting its distinct treatment of zero mass. Overlap of statistically significant findings was more modest (Jaccard 0.18–0.54; κ = 0.26–0.66), consistent with known differences in error structures and standard-error estimation across models and with dichotomous significance.

To avoid information loss from binary q-value thresholds, correlations between significance values were made and were found to be high between Gamma, Beta, two-part, and fractional logit specifications (typical r for signed Z ≈ 0.78–0.98; Spearman ρ for *p*-values ≈ 0.82–0.94), indicating consistent conclusions on both the direction and relative importance of predictors. These reporting choices align with guidance from the ASA and subsequent commentaries that recommend moving beyond strict “statistical significance” dichotomies [28]. Overall, conclusions about the presence, direction, and relative magnitude of associations are robust across modeling families. The analysis showed high concordance between models and corroborated the use of gamma regression with constant offset as the primary model (Table S6).

### 3.6. Structural Validity and Internal Consistency of Analysis 3 Questionnaires

Parallel analysis, for both the separate analysis for P.NET15 and P.NET19 and for the common core item set, with “specific symptoms”, indicated a one-factor solution (observed eigenvalues exceeded random-data eigenvalues only for the first factor). Exploratory factor analysis (minres) showed moderate–high loadings of core items on the first factor (loadings ≥ 0.40 are typically interpreted as salient, and loadings < 0.30 were omitted), and Cronbach’s α ranged from ~0.67 to 0.75 with corrected item–total correlations mostly ≥ 0.30, indicating acceptable internal consistency. These results are valid also for “specific symptoms”, with a strong loading on the first factor, indicating that, despite being assembled from module-specific items, it largely reflects the same latent dimension as the shared core (overall PanNET symptom burden). This supports its use as a pragmatic summary index, while interpreting it cautiously because it is not a formally validated scale (Tables S7–S12).

## 4. Discussion

Most studies evaluating HRQoL in GEP-NET patients include small number series of both gastrointestinal and pancreatic NETs or syndromic patients [29,30,31,32], and the effect of other available treatments for GEP-NETs is usually investigated, rather than surgery [33,34]. As in the present study, a general negative effect of older age and comorbidities on QoL and physical performance has already been reported [35,36,37], as well as a worsening of daily life activities after surgery for cancers of the hepato–pancreato-biliary tracts [38]. The absence of significant influence of gender in Analyses 1 and 2 partially deviates from previous published studies. Slightly higher global QoL results were reported in women (median 37.3%, IQR 15.2–59.4%) than in men (median 31.8%, IQR 8.7–59.9%) in 500 Polish cancer patients [39]. In contrast, other studies proved a non-significant [40] or worsening effect of female gender in GEP-NET patients [36,41,42,43].

In this observational single-center study, pancreatic function emerged as the most relevant clinical predictor variable, confirmed both as statistically significant results and as “a little” to “moderate” changes compared to the related MCIDs. Diabetes mellitus seems to be associated with worse global QoL outcomes (*p*-value < 0.001) and a worsening in functional and symptom scales, as previously reported in the diabetic population [44] and in the post-pancreatectomy population [45]. The present study also showed some positive correlations at univariable analysis between exocrine insufficiency and HRQoL outcomes. Patients who receive pancreatic enzyme replacement therapy feel a better HRQoL; malabsorption in these patients may be misdiagnosed, and exocrine insufficiency should always be tested after pancreatic resections [46].

Type of surgery has a clear impact on the postoperative pancreatic function impairment, as confirmed in a recent review [47]; new-onset diabetes and exocrine insufficiency were significantly higher after pancreatico-duodenectomy when compared to duodenum-preserving pancreatic head resection (DPPHR), and also after distal pancreatectomy when compared to central pancreatectomy. Positive correlations between parenchyma-sparing resections and HRQoL outcomes were previously demonstrated [48,49] and also confirmed by our study at univariable analysis as having a beneficial impact on fatigue and muscular performance.

Specific questionnaires investigating HRQoL in patients affected by PanNETs have been missing for a long time. Our group first reported on HRQoL determinants in PanNET patients who had undergone surgery, using other EORTC questionnaires [14]. The development of the new EORTC questionnaires P.NET19 and P.NET15, still in the validation phase, answered the need to investigate more specific issues of PanNET patients, as described by Ramage et al. [15]. The choice of unifying two populations with sometimes extremely different clinical scenarios, such as Gas/NF patients, was questioned while performing this study; however, the module team made separate reliability analyses, and good internal consistency was confirmed. The authors [15] created the domains purely based on clinical background, and in the absence of specific EORTC guidelines, we followed that study to arrange Analysis 3.

Analysis 3 was theorized to provide reliable results, with fewer items compared to previous questionnaires, from a consistent series of PanNET patients. The most frequently affected outcome was muscle energy at univariable analysis, with gut being the most affected outcome at multivariable analysis. In addition to the expected impact of age, standard pancreatic resections (related to an increased incidence of diabetes mellitus) showed a negative association with muscle energy, thus confirming previous clinical data. Apart from the above-mentioned exocrine insufficiency, Gas/NF diagnosis was the most important predictor negatively affecting QoL outcomes at both univariable and multivariable analyses. This finding may be in line with the natural history of NF-PanNETs and gastrinomas when compared with the excellent prognosis and HRQoL of insulinoma patients after treatment [12].

Strengths of the present study include its novelty, since, to our knowledge, no previous studies investigated HRQoL in PanNET patients after surgery using the EORTC questionnaires P.NET15 and P.NET19. Moreover, 11 years (median) between surgery and the administration of questionnaires represent a sufficient FU time to draw reliable conclusions regarding the long-term impact of surgery on HRQoL in these slow-growing neoplasms.

However, there are also some limitations. First, this is a single-center study with an observational design with a sample of about 100 patients, limiting precision and the scope for fully adjusted multivariable models without overfitting. Second, the cross-sectional design precludes causal claims (but allows hypothesis generation), and MCIDs reflect cross-sectional between-person contrasts rather than within-person change. The two new EORTC questionnaires are still in the validation phase, and no specific EORTC guidelines are available for the interpretation of the related items and domains. Modeling choices can affect inference: two-part models depend on the hurdle specification and sometimes yield sparse cells; bootstrap *p*-values may be conservative in small samples, whereas Wald *p*-values can be anti-conservative if model assumptions are strained; Beta/FracLogit analyses require careful handling of boundary values; and our rescaling and offset sensitivity analyses mitigate but cannot eliminate this concern. Multiple testing remains an issue even with FDR control within the outcome. Moreover, the lack of studies and standardization makes HRQoL investigations a challenge in this subset of patients. Furthermore, HRQoL measurement combined two disease-specific instruments (P.NET15 and P.NET19), which motivated the construction of a “Specific symptoms” composite that improves completeness but may introduce construct heterogeneity. Consistent with COSMIN guidance [24], reliability/validity analyses were therefore restricted to relevant subgroups, with the evaluation of a small common-item core, but pooled α for the composite variable should be interpreted cautiously. These limitations are aligned with STROBE guidance to discuss potential bias, imprecision, and generalizability.

## 5. Conclusions

In conclusion, PanNET patients showed a good HRQoL after tailored pancreatic surgery, even in the presence of active disease. Considering the new EORTC-specific questionnaires P.NET15 and P.NET19, old age, standard resections, diabetes mellitus, and Gas/NF diagnosis showed a possible negative impact on HRQoL outcomes. Routine evaluation of HRQoL in clinical practice and further studies on this topic may be useful to refine the therapeutic choices and the FU strategies in PanNET patients.

## Figures and Tables

**Table 1:** Clinical features of the study population (n = % = 100).

At the Time of Surgery		
Gender, n	Female	60
	Male	40
Type of NET, n	Gastrinoma/NF ^a^	68
	insulinoma	32
Tumor Grade, n	G2	23
	G1	72
	N/A	5
Type of Surgery, n	Standard pancreatic resection ^b^	54
	Parenchyma-sparing resection ^c^	46
MEN1 syndrome, n	Yes	17
	No	83
**At the time of the study**		
Time from surgery (months)	Median (IQR)	133 (52−219)
Age at questionnaire (years)	Median (IQR)	65 (55−73)
Other diseases, n	Multiple	63
	Single	23
	No	14
Tumor burden, n	Distant metastases ^d^	5
	Local recurrence	6
	Non-evidence of disease	89
Active disease, n	Yes	21
	No	79
Other treatments, n	Yes	15
	No	85
Pancreatic function, n	Diabetes mellitus	29
	Exocrine insufficiency	4
	Diabetes mellitus and Exocrine insufficiency	6
	Normal	61

NF, non-functioning. NET, neuroendocrine tumor. N/A, missing data. MEN1, multiple endocrine neoplasia type 1. IQR, interquartile range. ^a^ 63 non-functioning tumors. ^b^ In total: one total pancreatectomy, eight pancreatico-duodenectomies, one pancreatico-duodenectomy and spleen-preserving left pancreatectomy, 32 left pancreatectomies, and 12 spleen-preserving left pancreatectomies. ^c^ In total: 31 enucleations, 11 central pancreatectomies, and 4 duodenum-preserving pancreatic head resections. ^d^ Two out of five patients had liver metastases at diagnosis. Values are n (%) unless otherwise indicated.

## Data Availability

The original contributions presented in this study are included in the article/Supplementary Material. Further inquiries (concerning raw/anonymized domain scores) can be directed to the corresponding author.

## Supplementary Materials

The following supporting information can be downloaded at: https://www.mdpi.com/article/10.3390/cancers17193205/s1. Table S1: Distribution and median of functional and symptom outcome variables of EORTC QLQ-C30 (Analysis 1) and first 10 items of EORTC QLQ-P.NET15/P.NET19 (Analysis 2); Table S2: Results of univariable Gamma model regression (Analysis 1) regarding predictor variables and quality of life outcomes. Data are reported as average marginal effect, 95% CI, and *p*-value; Table S3: Results of univariable Gamma model regression (Analysis 2) regarding predictor variables and quality of life outcomes. Data are reported as average marginal effect, 95% CI, and *p*-value; Table S4: Distribution and median of outcome variables of EORTC QLQ-P.NET15 and EORTC QLQ-P.NET19 (Analysis 3); Table S5: Results of univariable Gamma model regression (Analysis 3) regarding predictor variables and quality of life outcomes. Data are reported as average marginal effect, 95% CI, and *p*-value. Figure S1. Forest plot for multivariable analysis of Analysis 1. Figure S2. Forest plot for multivariable analysis of Analysis 2. Figure S3. Forest plot for multivariable analysis of Analysis 3. Table S6. Model comparison. Table S7. Structural validity and internal consistency for Analysis 3 and specific symptoms. Insulinoma (PNET.19) internal consistency. Table S8. Structural validity and internal consistency for Analysis 3 and specific symptoms. Parallel exploratory factor analysis for Insulinoma (PNET.19). Table S9. Structural validity and internal consistency for Analysis 3 and specific symptoms. Gastrinoma/Non-functioning (PNET.15) internal consistency. Table S10. Structural validity and internal consistency for Analysis 3 and specific symptoms. Parallel exploratory factor analysis for Gastrinoma/Non-functioning (PNET.15). Table S11. Structural validity and internal consistency for Analysis 3 and specific symptoms. Analysis of the common symptoms set (internal consistency). Table S12. Structural validity and internal consistency for Analysis 3 and specific symptoms. Analysis of the common symptoms set (parallel factor analysis).

## Abbreviations

The following abbreviations are used in this manuscript:

PanNET	pancreatic neuroendocrine tumor
NF-PanNET	non-functioning pancreatic neuroendocrine tumor
MEN1	multiple endocrine neoplasia type 1
HRQoL	health-related quality of life
GEP-NET	gastro–entero-pancreatic neuroendocrine tumor
EORTC	European Organization for Research and Treatment of Cancer
FU	follow-up
Gas/NF-PanNET	gastrinoma/non-functioning pancreatic neuroendocrine tumor
AME	Average Marginal Effect
MCIDs	minimal clinically important differences
FDR	false-discovery rate
PF2	physical functioning
EF	emotional functioning
GI	Gastrointestinal
DPPHR	duodenum-preserving pancreatic head resection
