# Supplementary material for: Quality of Life After Pancreatic Surgery for Neuroendocrine Tumors of the Pancreas: Observational Study of Long-Term Outcomes
Source: Cancers (Basel). 2025 Oct 1;17(19):3205. doi: 10.3390/cancers17193205 (PMC12524290; doi:10.3390/cancers17193205)
Supplement: Supplementary file 1 [file cancers-17-03205-s001.zip › cancers-3827581-supplementary.pdf]

# Quality of life after Pancreatic Surgery for Neuroendocrine Tumors of the Pancreas: Observational Study of Long-term Outcomes

**Anna Caterina Milanetto[1,2], Claudia Armellin[3], Daniele Gasparini[4], Giulia Lorenzoni[4], Claudio Pasquali[1]**

[1]Pancreatic and Digestive Endocrine Surgery, Department of Surgery, Oncology and Gastroenterology, University of Padua, via Giustiniani 2, 35128 Padua, Italy. claudio.pasquali@unipd.it
[2]UniCamillus International Medical University in Rome, via di Sant'Alessandro 8, 00131 Rome, Italy. annacaterina.milanetto@unicamillus.org
[3]Department of General Surgery, Policlinico di Abano Terme, piazza Cristoforo Colombo 1, 35031 Abano Terme (PD), Italy. carmellin@casacura.it
[4]Unit of Biostatistics, Epidemiology and Public Health, Department of Cardiac, Thoracic, Vascular Sciences and Public Health, University of Padova, via Loredan 18, 35131 Padua, Italy. giulia.lorenzoni@unipd.it, daniele.gasparini@ubep.unipd.it

**Corresponding author:** annacaterina.milanetto@unicamillus.org

## Supplementary Materials - Index

### Supplementary Figures and Tables

| | |
|---|---|
| Table S1 | *pag. 3* |
| Table S2 | *pag. 4-5* |
| Table S3 | *pag. 6-7* |
| Table S4 | *pag. 8* |
| Table S5 | *pag. 9-10* |
| Figure S1 | *pag. 11* |
| Figure S2 | *Pag. 12* |
| Figure S3 | *Pag. 12* |
| Table S6 | *Pag. 14* |
| Table S7 | *Pag. 15* |
| Table S8 | *Pag. 16* |

| | |
|---|---|
| Table S9 | *Pag. 17* |
| Table S10 | *Pag. 18* |
| Table S11 | *Pag. 19* |
| Table S12 | *Pag. 20* |

**Supplementary Figures and Tables**

**Table S1**. Distribution and median of functional and symptom outcome variables of EORTC QLQ-C30 (Analysis 1) and first 10 items of EORTC QLQ-P.NET15/P.NET19 (Analysis 2)

| **Analysis 1** | | | | **Analysis 2** | | | |
|---|---|---|---|---|---|---|---|
| **Outcome variable (missing data)** | Median Value (%) | IQR (%) | Min-Max (%) | **Outcome variable (missing data)** | Median Value (%) | IQR (%) | Min-Max (%) |
| **Global QoL** | 83 | 67-85 | 33-100 | **Global QoL** | 83 | 67-85 | 25-100 |
| **Functional Scales**[a] | | | | **Functional Scales**[a] | | | |
| Physical Functioning | 93 | 80-100 | 26-100 | Physical Functioning | 93 | 80-100 | 26-100 |
| Role functioning | 100 | 83-100 | 16-100 | Role functioning | 100 | 83-100 | 0-100 |
| Emotional Functioning | 83 | 75-100 | 33-100 | Emotional Functioning | 87 | 78-100 | 33-100 |
| Cognitive Functioning | 100 | 83-100 | 16-100 | Cognitive Functioning | 100 | 83-100 | 16-100 |
| Social functioning (1%) | 100 | 100-100 | 16-100 | Social functioning (1%) | 100 | 100-100 | 16-100 |
| **Symptom Scales**[b] | | | | **Symptom Scales**[b] | | | |
| Fatigue | 19 | 0-33 | 0-55 | Fatigue | 13 | 0-33 | 0-100 |
| Pain | 0 | 0-17 | 0-66 | Pain | 0 | 0-17 | 0-66 |
| Dyspnoea (1%) | 0 | 0-0 | 0-66 | Dyspnoea (1%) | 0 | 0-0 | 0-100 |
| Sleep Difficulties | 0 | 0-33 | 0-66 | Sleep Difficulties | 0 | 0-33 | 0-100 |
| Financial Difficulties (1%) | 0 | 0-0 | 0-66 | Financial Difficulties (1%) | 0 | 0-0 | 0-66 |
| Appetite Loss | 0 | 0-0 | 0-66 | Upper-GI Symptoms | 0 | 0-8 | 0-41 |
| Nausea/Vomiting | 0 | 0-0 | 0-66 | Lower-GI Symptoms | 11 | 6-22 | 0-55 |
| Constipation | 0 | 0-33 | 0-66 | Sweating | 0 | 0-0 | 0-100 |
| Diarrhoea | 0 | 0-0 | 0-100 | Body Image (2%) | 0 | 0-0 | 0-66 |

**Legend**. QoL, quality of life. IQR, interquartile range. GI, gastrointestinal.
(%) percentage of missing data is reported in brackets.
[a]Scores range from 0 to 100, with a higher score representing a higher level of function.
[b]Scores range from 0 to 100, with a higher score representing a higher level of symptoms.

**Table S2**. Results of univariable Gamma model regression (Analysis 1) regarding predictor variables and quality of life outcomes. Data are reported as average marginal effect, 95% CI, and *p value.*

| | | | | | **Functional Scales** | | | | | | **Symptom Scales** | | | | | |
|---|---|---|---|---|---|---|---|---|---|---|---|---|---|---|---|---|
| | | **Global QoL** | | | **Physical Functioning** | | | **Emotional Functioning** | | | **Pain** | | | **Fatigue** | | |
| | | **AME [95% CI]** | ***p* value** | ***q value (FDR)*** | **AME [95% CI]** | ***p* value** | ***q value (FDR)*** | **AME [95% CI]** | ***p* value** | ***q value (FDR)*** | **AME [95% CI]** | ***p* value** | ***q value (FDR)*** | **AME [95% CI]** | ***p* value** | ***q value (FDR)*** |
| Age | | -0.38 [-0.66; -0.10] | ***0.007*** | *0.058* | -0.45 [-0.67; -0.23] | ***0.00007*** | ***0.001*** | -0.10 [-0.32; 0.12] | *0.367* | *0.875* | 0.20 [-0.05; 0.45] | *0.113* | *0.592* | 0.18 [-0.08; 0.43] | *0.167* | *0.317* |
| Gender | Female | -2.36 [-10.72; 6.00] | *0.580* | *0.796* | -6.06 [-12.89; 0.78]§ | *0.082* | *0.350* | -1.87 [-8.44; 4.69] | *0.576* | *0.875* | 1.44 [-5.41; 8.29] | *0.680* | *0.826* | -0.92 [-8.40; 6.65] | *0.808* | *0.857* |
| | Male | | | | | | | | | | | | | | | |
| Active disease | Yes | 4.76 [-5.60; 15.12] | *0.368* | *0.642* | 2.52 [-5.76; 10.80] | *0.551* | *0.781* | 0.05 [-7.80; 7.92] | *0.989* | *0.989* | -2.61 [-10.16; 4.95] | *0.498* | *0.826* | 1.39 [-7.90; 10.68] | *0.769* | *0.857* |
| | No | | | | | | | | | | | | | | | |
| Other diseases | Multiple | -12.23 [-25.22; 0.75]§ | *0.065* | *0.261* | -14.13 [-24.84; -3.41]§ | ***0.0097*** | *0.055* | -4.23 [-13.96; 5.49] | *0.394* | *0.875* | 8.28 [1.27; 15.29]§ | ***0.021*** | *0.351* | 11.77 [4.81; 18.73]§ | ***0.001*** | ***0.016*** |
| | Single | -2.66 [-17.77; 12.44] | *0.729* | *0.885* | -9.30 [-21.46; 2.87]§ | *0.134* | *0.4355* | -0.67 [-11.89; 10.55] | *0.906* | *0.989* | 2.12 [-4.78; 9.01] | *0.547* | *0.826* | 7.95 [-0.42; 16.33]§ | *0.062* | *0.177* |
| | No | | | | | | | | | | | | | | | |
| Type of NET | Gastrinoma /NF | -9.67 [-18.82; -0.51]§ | ***0.039*** | *0.218* | -2.71 [-9.92; 4.50] | *0.461* | *0.781* | -5.41 [-12.40; 1.58]§ | *0.129* | *0.875* | 4.82 [-1.68; 11.32] | *0.146* | *0.592* | 8.15 [1.51; 14.80]§ | ***0.016*** | *0.093* |
| | Insulinoma | | | | | | | | | | | | | | | |
| Type of surgery | Standard | -0.74 [-8.93; 7.44] | *0.858* | *0.912* | -0.27 [-6.95; 6.40] | *0.935* | *0.936* | -2.30 [-8.71; 4.11] | *0.481* | *0.875* | -1.84 [-8.86; 5.18] | *0.607* | *0.826* | 7.10 [0.11; 14.11]§ | ***0.046*** | *0.158* |
| | Parenchyma -sparing | | | | | | | | | | | | | | | |
| Pancreatic function | Diabetes mellitus | -15.15 [-23.63; -6.68]§ | ***0.0005*** | ***0.008*** | -10.36 [-17.55; -3.17]§ | ***0.005*** | ***0.040*** | -3.37 [-10.42; 3.67] | *0.347* | *0.875* | 7.61 [-2.17; 17.38]§ | *0.127* | *0.592* | 10.45 [1.00; 19.91]§ | ***0.030*** | *0.128* |
| | Exocrine insufficiency | 10.35 [-14.55; 35.24]§ | *0.415* | *0.642* | 7.51 [-11.68; 26.70]§ | *0.443* | *0.781* | 15.30 [-4.09; 34.69]§ | *0.122* | *0.875* | -5.31 [-12.95; 2.35]§ | *0.174* | *0.592* | -8.41 [-15.31; -1.53]§ | ***0.016*** | *0.093* |

| | | | | | | | | | | | | | | | | |
|---|---|---|---|---|---|---|---|---|---|---|---|---|---|---|---|---|
| | Diabetes mellitus and exocrine insufficiency | -13.96 [-29.42; 1.50]§ | *0.077* | *0.261* | -9.71 [-23.04; 3.62]§ | *0.153* | *0.435* | -4.14 [-17.27; 8.98] | *0.536* | *0.875* | 1.40 [-12.11; 14.90] | *0.839* | *0.869* | 18.77 [-7.11; 44.66]§ | *0.155* | *0.317* |
| | Normal | | | | | | | | | | | | | | | |
| Time from surgery | | -0.03 [-0.07; 0.01] | *0.163* | *0.462* | -0.01 [-0.05; 0.02] | *0.410* | *0.781* | -0.01 [-0.04; 0.02] | *0.456* | *0.875* | -0.01 [-0.04; 0.03] | *0.677* | *0.826* | -0.03 [-0.04; 0.03] | *0.857* | *0.857* |
| Other treatments | Yes | -5.20 [-16.04; 5.65]§ | *0.348* | *0.642* | -2.59 [-11.68; 6.50] | *0.577* | *0.781* | 0.42 [-8.57; 9.42] | *0.926* | *0.989* | -1.99 [-10.53; 6.53] | *0.646* | *0.826* | 4.63 [-7.34; 16.61] | *0.447* | *0.761* |
| | No | | | | | | | | | | | | | | | |

**Legend.** AME, average marginal effect. NET, neuroendocrine tumor. NF, nonfunctioning. QoL, quality of life.
§"A little" to "moderate" changes compared to the related minimal clinically important differences (MCIDs).

**Table S3.** Results of univariable Gamma model regression (Analysis 2) regarding predictor variables and quality of life outcomes. Data are reported as average marginal effect, 95% CI, and *p value*.

| | | **Functional Scales** | | | | | | | | | **Symptom Scales** | | | | | |
|---|---|---|---|---|---|---|---|---|---|---|---|---|---|---|---|---|
| | | **Global QoL** | | | **Physical Functioning** | | | **Emotional Functioning** | | | **Upper-GI Symptoms** | | | **Lower-GI Symptoms** | | |
| | | **AME [95% CI]** | ***p value*** | ***q value (FDR)*** | **AME [95% CI]** | **p value** | ***q value (FDR)*** | **AME [95% CI]** | ***p value*** | ***q value (FDR)*** | **AME [95% CI]** | ***p value*** | ***q value (FDR)*** | **AME [95% CI]** | ***p value*** | ***q value (FDR)*** |
| Age | | -0.38 [-0.66; -0.10] | ***0.006*** | *0.057* | -0.45 [-0.67; -0.23] | ***0.0007*** | ***0.001*** | -0.13 [-0.34; 0.08] | *0.225* | *0.766* | 0.03 [-0.08; 0.15] | *0.562* | *0.922* | 0.01 [-0.17; 0.19] | *0.942* | *0.942* |
| Gender | Female | -2.36 [-10.72; 6.00] | *0.580* | *0.795* | -6.06 [-12.89; 0.78] | *0.082* | *0.350* | -3.02 [-9.33; 3.30] | *0.348* | *0.799* | -0.73 [-4.18; 2.74] | *0.682* | *0.966* | -0.29 [-5.65; 5.07] | *0.915* | *0.942* |
| | Male | | | | | | | | | | | | | | | |
| Active disease | Yes | 4.76 [-5.60; 15.12] | *0.368* | *0.641* | 2.52 [-5.76; 10.80] | *0.551* | *0.781* | 0.07 [-7.47; 7.61] | *0.985* | *0.996* | 2.34 [-2.71; 7.40] | *0.363* | *0.922* | -1.44 [-7.49; 4.60] | *0.639* | *0.835* |
| | No | | | | | | | | | | | | | | | |
| Other diseases | Multiple | -12.23 [-25.22; 0.75] | *0.064* | *0.261* | -14.13 [-24.84; -3.41] | ***0.009*** | *0.055* | -6.10 [-15.56; 3.36] | *0.206* | *0.766* | 3.12 [-0.71; 6.95] | *0.110* | *0.922* | 4.28 [-2.15; 10.71] | *0.191* | *0.835* |
| | Single | -2.67 [-17.77; 12.44] | *0.729* | *0.885* | -9.30 [-21.46; 2.87] | *0.134* | *0.434* | -2.86 [-13.71; 8.00] | *0.605* | *0.858* | -1.58 [-4.87; 1.72] | *0.347* | *0.922* | 3.38 [-4.31; 11.08] | *0.389* | *0.835* |
| | No | | | | | | | | | | | | | | | |
| Type of NET | Gastrinoma/NF | -9.67 [-18.82; -0.51] | ***0.038*** | *0.218* | -2.71 [-9.92; 4.50] | *0.461* | *0.781* | -6.09 [-12.80; 0.61] | *0.074* | *0.766* | 1.71 [-1.56; 4.97] | *0.305* | *0.922* | 2.21 [-3.24; 7.65] | *0.426* | *0.835* |
| | Insulinoma | | | | | | | | | | | | | | | |
| Type of surgery | Standard | -0.74 [-8.93; 7.44] | *0.858* | *0.912* | -0.27 [-6.95; 6.40] | *0.935* | *0.935* | -2.78 [-8.91; 3.36] | *0.375* | *0.799* | 0.05 [-3.23; 3.33] | *0.976* | *0.985* | 2.33 [-2.92; 7.57] | *0.384* | *0.835* |
| | Parenchyma-sparing | | | | | | | | | | | | | | | |
| Pancreatic function | Diabetes mellitus | -15.15 [-23.63; -6.68] | ***0.0004*** | ***0.007*** | -10.36 [-17.55; -3.17] | ***0.004*** | ***0.040*** | -5.00 [-11.71; 1.70] | *0.143* | *0.766* | 1.05 [-2.64; 4.75] | *0.575* | *0.922* | 6.16 [-0.56; 12.88] | *0.072* | *0.835* |
| | Exocrine insufficiency | 10.35 [-14.55; 35.24] | *0.415* | *0.641* | 7.51 [-11.68; 26.70] | *0.442* | *0.781* | 13.77 [-4.60; 32.14] | *0.141* | *0.766* | -0.14 [-7.33; 7.04] | *0.968* | *0.985* | 3.73 [-10.80; 18.26] | *0.614* | *0.835* |
| | Diabetes mellitus and exocrine insufficiency | -13.96 [-29.42; 1.50] | *0.076* | *0.261* | -9.71 [-23.04; 3.62] | *0.153* | *0.434* | -5.12 [-17.64; 7.40] | *0.423* | *0.799* | 7.61 [-7.12; 22.33] | *0.311* | *0.922* | 5.80 [-7.68; 19.28] | *0.399* | *0.835* |
| | Normal | | | | | | | | | | | | | | | |
| Time from surgery | | -0.03 [-0.07; 0.01] | *0.163* | *0.462* | -0.01 [-0.05; 0.02] | *0.409* | *0.781* | -0.01 [-0.04; 0.02] | *0.394* | *0.799* | 0.01 [-0.01; 0.02] | *0.597* | *0.922* | 0.02 [-0.01; 0.04] | *0.192* | *0.835* |

**Legend.** AME, average marginal effect. GI, gastrointestinal. NET, neuroendocrine tumor. NF, nonfunctioning. QoL, quality of life.

**Table S4**. Distribution and median of outcome variables of EORTC QLQ-P.NET15 and EORTC QLQ-P.NET19 (Analysis 3)

| **Outcome variable (missing data)** | Median Value (%) | IQR (%) | Min-Max (%) |
|---|---|---|---|
| Gut | 8 | 0-25 | 0-58 |
| Muscle/Energy | 17 | 0-33 | 0-100 |
| Weight/Food Restrictions (1%) | 0 | 0-17 | 0-66 |
| Sweating | 0 | 0-0 | 0-100 |
| Frustration | 0 | 0-33 | 0-66 |
| Dyspepsia/ulceration – Gas/NF symptoms (33%) | 0 | 0-11 | 0-55 |
| Low Blood Glucose/Neurological Symptoms – HYPO (68%) | 0 | 0-4 | 1-18 |
| Itching (33%) | 0 | 0-33 | 0-66 |
| Nocturia (33%) | 0 | 0-33 | 0-66 |
| Specific Symptoms (1%) | 4 | 0-8 | 0-32 |

**Legend**. IQR, interquartile range. Gas/NF symptoms, nonfunctioning pancreatic neuroendocrine tumor and gastrinoma-related symptoms. HYPO, Low blood glucose/neurological symptoms.
(%) percentage of missing data is reported in brackets.
Scores range from 0 to 100, with a higher score representing a higher level of symptoms.

**Table S5**. Results of univariable Gamma model regression (Analysis 3) regarding predictor variables and quality of life outcomes. Data are reported as average marginal effect, 95% CI, and *p value*.

| | | **Gut** | | | **Muscle/energy** | | | **Weight and Food restrictions** | | | **Sweating** | | | **Frustration** | | |
|---|---|---|---|---|---|---|---|---|---|---|---|---|---|---|---|---|
| | | **AME [95% CI]** | ***p value*** | ***q value (FDR)*** | **AME [95% CI]** | ***p value*** | ***q value (FDR)*** | **AME [95% CI]** | ***p value*** | ***q value (FDR)*** | **AME [95% CI]** | ***p value*** | ***q value (FDR)*** | **AME [95% CI]** | ***p value*** | ***q value (FDR)*** |
| Age | | -0.08 [-0.30; 0.14] | *0.490* | *0.758* | 0.32 [0.01; 0.63] | ***0.042*** | *0.090* | -0.05 [-0.22; 0.11] | *0.492* | *0.760* | 0.13 [-0.15; 0.41] | *0.368* | *0.521* | 0.50 [0.05; 0.94] | ***0.027*** | *0.078* |
| Type of NET | Gastrinoma/NF | 3.29 [-3.24; 9.82] | *0.323* | *0.758* | 10.39 [2.82; 17.96] | ***0.007*** | ***0.030*** | 4.14 [-0.02; 8.29] | *0.051* | *0.435* | -1.63 [-10.35; 7.09] | *0.714* | *0.759* | 8.29 [1.21; 15.37] | ***0.021*** | *0.073* |
| | Insulinoma | | | | | | | | | | | | | | | |
| Active disease | Yes | -0.36 [-8.07; 7.34] | *0.926* | *0.926* | -1.17 [-10.89; 8.55] | *0.813* | *0.910* | 5.73 [-2.31; 13.76] | *0.162* | *0.462* | -7.65 [-13.75; -1.55] | ***0.013*** | ***0.047*** | 0.14 [-8.90; 9.17] | *0.976* | *0.989* |
| | No | | | | | | | | | | | | | | | |
| Pancreatic function | Diabetes mellitus | 5.52 [-2.43; 13.47] | *0.173* | *0.758* | 15.88 [3.21; 28.54] | ***0.014*** | ***0.040*** | -3.11 [-7.58; 1.37] | *0.174* | *0.462* | 3.82 [-6.60; 14.25] | *0.472* | *0.617* | 10.51 [-0.92; 21.93] | *0.071* | *0.152* |
| | Exocrine insufficiency | 4.74 [-14.01; 23.49] | *0.620* | *0.853* | -9.31 [-15.89; -2.71] | ***0.005*** | ***0.030*** | -8.00 [-11.55; -4.45] | ***0.0000*** | ***0.0001*** | -8.54 [-13.52; -3.57] | ***0.0007*** | ***0.003*** | -7.44 [-11.13; -3.74] | ***0.0000*** | ***0.0004*** |
| | Diabetes mellitus and exocrine insufficiency | 6.57 [-10.29; 23.43] | *0.444* | *0.758* | 19.28 [-10.45; 49.01] | *0.203* | *0.384* | 2.45 [-11.06; 15.95] | *0.722* | *0.883* | -3.16 [-14.01; 7.69] | *0.568* | *0.690* | 8.90 [-13.34; 31.13] | *0.432* | *0.613* |
| | Normal | | | | | | | | | | | | | | | |
| Type of surgery | Standard | 2.31 [-4.07; 8.67] | *0.478* | *0.758* | 10.40 [1.73; 19.06] | ***0.018*** | ***0.045*** | 0.35 [-4.31; 5.00] | *0.883* | *0.883* | -4.14 [-12.25; 3.98] | *0.318* | *0.491* | 4.11 [-3.47; 11.70] | *0.288* | *0.489* |
| | Parenchyma-sparing | | | | | | | | | | | | | | | |
| Time from surgery | | 0.02 [-0.01; 0.05] | *0.173* | *0.758* | -0.004 [-0.04; 0.04] | *0.855* | *0.910* | -0.003 [-0.03; 0.02] | *0.788* | *0.883* | -0.03 [-0.08; 0.02] | *0.180* | *0.307* | 0.02 [-0.02; 0.06] | *0.258* | *0.487* |
| | | **HYPO[a]** | | | **Gas/NF symptoms[a]** | | | **Itching** | | | **Nocturia** | | | **Specific symptoms** | | |
| | | **AME [95% CI]** | ***p value*** | ***q value (FDR)*** | **AME [95% CI]** | ***p value*** | ***q value (FDR)*** | **AME [95% CI]** | ***p value*** | ***q value (FDR)*** | **AME [95% CI]** | ***p value*** | ***q value (FDR)*** | **AME [95% CI]** | ***p value*** | ***q value (FDR)*** |
| Age | | -0.01 [-0.15; 0.13] | *0.903* | *0.903* | 0.10 [-0.11; 0.28] | *0.366* | *0.566* | 0.10 [-0.18; 0.37] | *0.475* | *0.673* | 1.20 [0.22; 2.19] | ***0.016*** | *0.095* | 0.08 [0.00; 0.17] | *0.055* | *0.234* |

| | | | | | | | | | | | | | | | | |
|---|---|---|---|---|---|---|---|---|---|---|---|---|---|---|---|---|
| Type of NET | Gastrinoma/NF | -3.28 [-5.67; -0.90] | ***0.006*** | ***0.038*** | 7.52 [3.50; 11.54] | ***0.0002*** | ***0.004*** | 9.83 [4.67; 14.98] | ***0.0001*** | ***0.001*** | 18.28 [11.88; 24.68] | ***0.0000*** | ***0.0000*** | 2.39 [0.14; 4.64] | ***0.037*** | *0.211* |
| | Insulinoma | | | | | | | | | | | | | | | |
| Active disease | Yes | 1.68 [-6.21; 9.58] | *0.675* | *0.903* | -4.52 [-9.74; 0.70] | *0.089* | *0.319* | -0.88 [-9.84; 8.08] | *0.847* | *0.900* | 0.25 [-13.08; 13.58] | *0.971* | *0.971* | -0.36 [-3.23; 2.51] | *0.805* | *0.855* |
| | No | | | | | | | | | | | | | | | |
| Pancreatic function | Diabetes mellitus | 4.95 [-3.28; 13.18] | *0.238* | *0.298* | 4.03 [-2.54; 10.60] | *0.229* | *0.478* | -6.64 [-15.40; 2.13] | *0.137* | *0.468* | 3.14 [-9.57; 15.84] | *0.628* | *0.970* | 2.60 [-0.47; 5.66] | *0.096* | *0.273* |
| | Exocrine insufficiency | | | | 0.07 [-9.23; 9.37] | *0.987* | *0.987* | -13.38 [-20.90; -5.84] | ***0.0005*** | ***0.002*** | -17.24 [-24.73; -9.74] | ***0.0000*** | ***0.0000*** | -1.91 [-5.30; 1.21] | *0.229* | *0.446* |
| | Diabetes mellitus and exocrine insufficiency | 11.88 [-22.27; 46.03] | *0.495* | *0.962* | 9.72 [-10.43; 29.88] | *0.344* | *0.566* | -6.91 [-19.33; 5.52] | *0.276* | *0.493* | 9.03 [-20.76; 38.82] | *0.552* | *0.939* | 6.14 [-3.11; 15.39] | *0.193* | *0.446* |
| | Normal | | | | | | | | | | | | | | | |
| Type of surgery | Standard | 0.97 [-3.00; 4.93] | *0.632* | *0.903* | -0.14 [-6.21; 5.93] | *0.964* | *0.987* | 0.33 [-8.16; 8.82] | *0.939* | *0.939* | -10.05 [-25.05; 4.94] | *0.188* | *0.534* | 0.35 [-2.07; 2.77] | *0.775* | *0.855* |
| | Parenchyma-sparing | | | | | | | | | | | | | | | |
| Time from surgery | | 0.001 [-0.02; 0.02] | *0.879* | *0.903* | 0.02 [-0.01; 0.05] | *0.253* | *0.478* | 0.02 [-0.02; 0.07] | *0.256* | *0.493* | 0.08 [0.01; 0.17] | ***0.032*** | *0.136* | 0.01 [-0.00; 0.02] | *0.091* | *0.273* |

**Legend**. AME, average marginal effect. Gas/NF symptoms, nonfunctioning pancreatic neuroendocrine tumor and gastrinoma-related symptoms. HYPO, Low blood glucose/neurological symptoms. NET, neuroendocrine tumor. NF, nonfunctioning.

N/A refers to results non calculated due to lack of data.

[a]Subgroup analyses regarding HYPO (n = 32) and Gas/NF symptoms (n = 68).

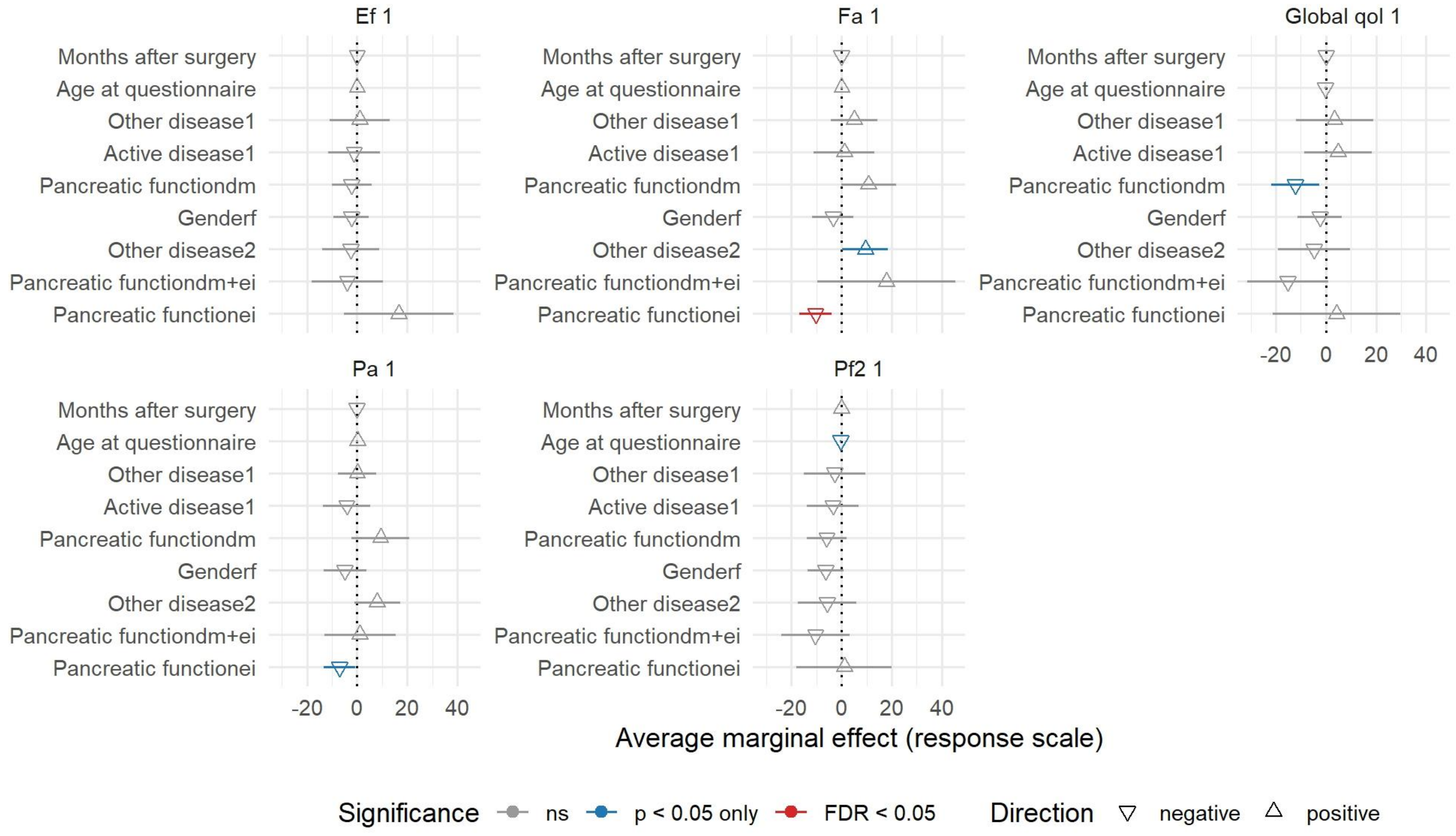


**Figure S1.** Forest plot for multivariable analysis of Analysis 1. EF, emotional functioning. FA, fatigue. QoL, quality of life. PA, pain. PF2, physical functioning. FDR, false-discovery rate. F, female. DM, diabetes mellitus. EI, exocrine insufficiency.

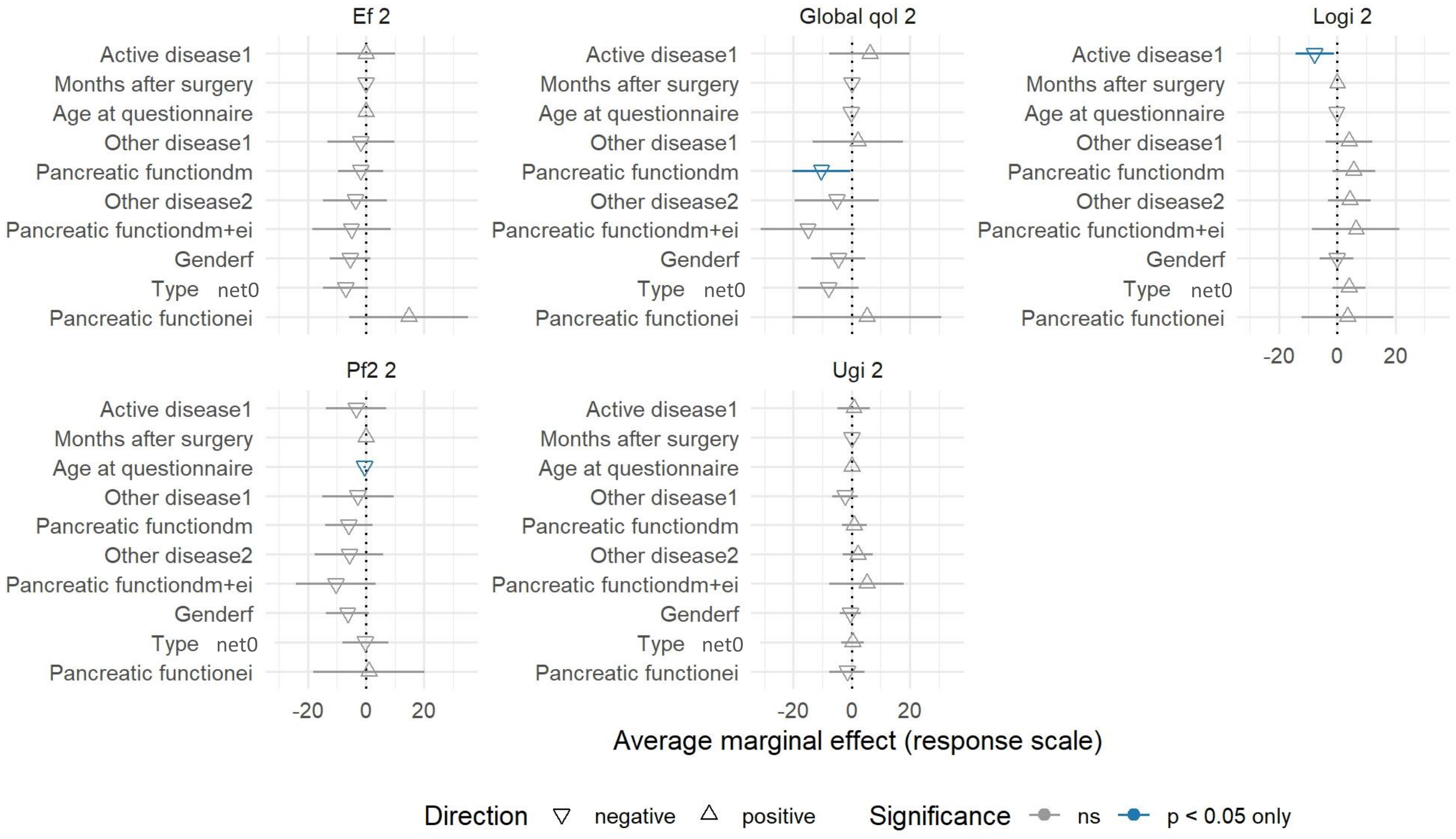


**Figure S2.** Forest plot for multivariable analysis of Analysis 2. EF, emotional functioning. QoL, quality of life. PF2, physical functioning. LOGI, lower gastrointestinal symptoms. UGI, upper gastrointestinal symptoms. NET, neuroendocrine tumor. DM, diabetes mellitus. EI, exocrine insufficiency.

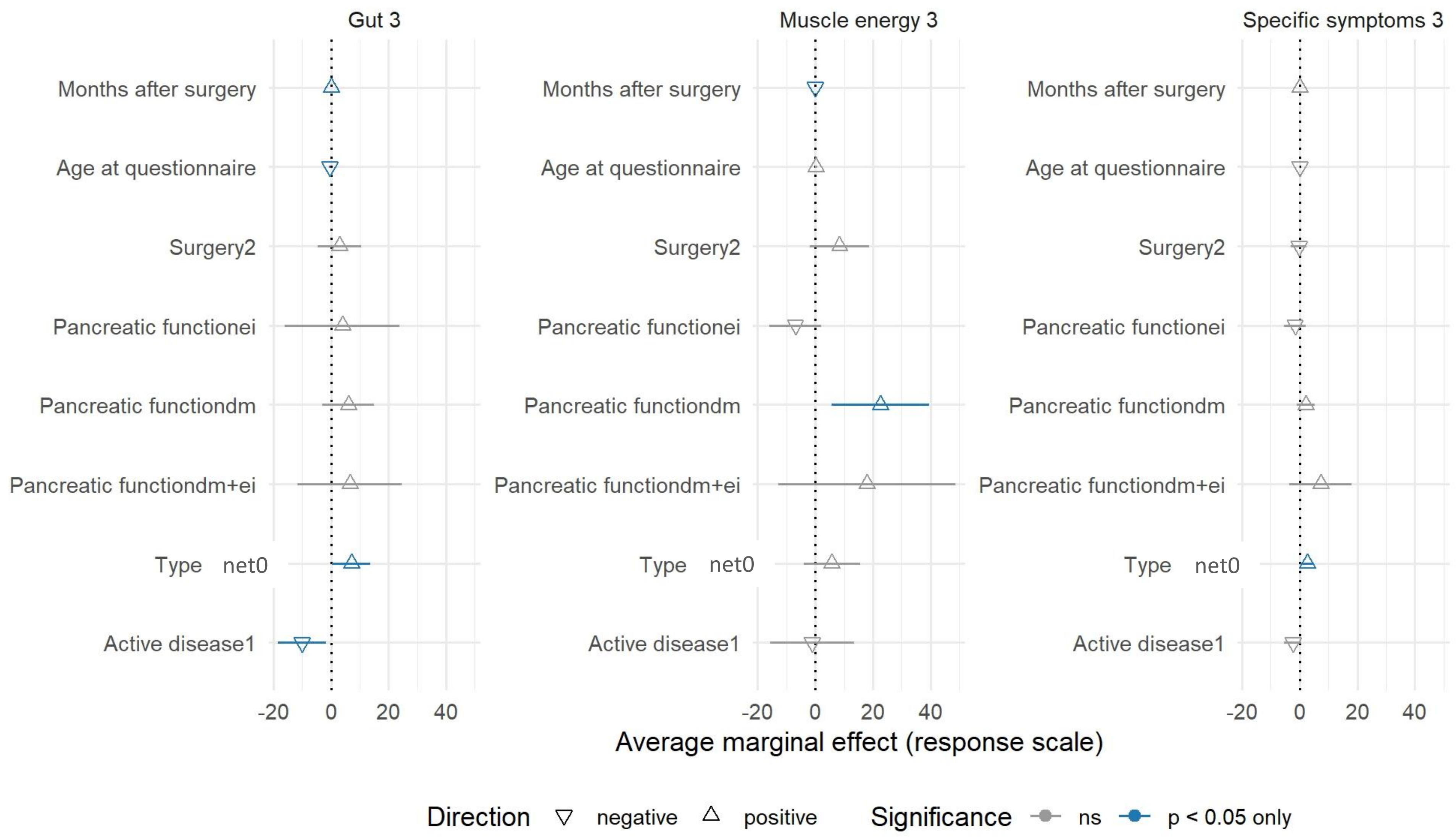


**Figure S3.** Forest plot for multivariable analysis of Analysis 3. NET, neuroendocrine tumor. DM, diabetes mellitus. EI, exocrine insufficiency.

**Table S6.** Model comparison.

| Model pair | N overlap | Cor effect | Sign agree | Jaccard sig | Kappa sig | corZ median | rhoP median |
|---|---|---|---|---|---|---|---|
| Beta vs Gamma | 674 | 0.9866 | 0.9733 | 0.2188 | 0.3284 | 0.8474 | 0.8291 |
| Beta vs FracLogit | 674 | 0.9859 | 0.9763 | 0.1782 | 0.2648 | 0.7871 | 0.8407 |
| Gamma vs FracLogit | 674 | 0.9999 | 0.997 | 0.5347 | 0.6605 | 0.9776 | 0.9432 |
| Gamma vs TwoPart | 674 | 0.94 | 0.9288 | 0.4783 | 0.6186 | 0.8871 | 0.8652 |
| Beta vs TwoPart | 674 | 0.9283 | 0.9199 | 0.375 | 0.5253 | 0.8713 | 0.8243 |
| FracLogit vs TwoPart | 674 | 0.9396 | 0.9258 | 0.4592 | 0.592 | 0.905 | 0.8511 |

**Legend. Model pair**: modeling families compared. **N overlap**: number of outcome-predictor contrasts. **Cor effect**: Pearson correlation between AMEs. **Sign agree**: proportion of contrasts where the two AMEs have the same sign. **Jaccard sig**: Jaccard index for FDR-adjusted $q<0.05$ contrasts. **Kappa sig**: Cohen's κ for $q<0.05$ vs $q \geq 0.05$. **corZ median**: median of the Pearson correlation between signed Z-scores. **rhoP median**: median of the Spearman correlation between *p* values.

**Table S7.** Structural validity and internal consistency for analysis 3 and specific symptoms. Insulinoma (PNET.19) internal consistency.

| Raw alpha | Std alpha | Average r | S/N |
|---|---|---|---|
| 0.6676 | 0.7481 | 0.3311 | 2.97 |

| item | n | mean | sd | Raw r | r drop | Alpha if deleted raw | Alpha if deleted std |
|---|---|---|---|---|---|---|---|
| Gut 3 | 29 | 14.66 | 19.24 | 0.7331 | 0.5264 | 0.5735 | 0.7048 |
| Muscle energy 3 | 29 | 12.64 | 17.62 | 0.8082 | 0.6637 | 0.5199 | 0.6456 |
| Weight food restriction 3 | 29 | 4.598 | 8.793 | 0.38 | 0.2486 | 0.6698 | 0.755 |
| Sweating 3 | 29 | 11.49 | 25.63 | 0.6759 | 0.3332 | 0.6954 | 0.7576 |
| Frustration 3 | 29 | 5.747 | 15.61 | 0.5653 | 0.354 | 0.64 | 0.738 |
| Ipo 3 | 29 | 3.879 | 5.448 | 0.7071 | 0.6585 | 0.6329 | 0.6494 |

**Legend. Raw alpha:** scale-dependent α. **Std alpha:** standardized α, unit-free. **Average r:** average inter-item correlation. **S/N**: signal-to-noise ratio (α/(1–α)). **N:** sample size (**n**). **SD**: Standard Deviation. **Raw r**: item–total correlation. **r drop:** corrected item–total correlation, excluding the item itself. **Alpha if deleted**: α if that item were removed; raw/standardized.

**Table S8.** Structural validity and internal consistency for analysis 3 and specific symptoms. Parallel exploratory factor analysis for Insulinoma (PNET.19)

Parallel analysis suggests that the number of factors = 1 and the number of components = NA
Parallel analysis indicated a 1-factor solution (observed eigenvalues exceeded random-data eigenvalues only for the first factor).

Loadings:

| | MR1 |
|---|---|
| gut 3 | 0.564 |
| muscle energy 3 | 0.834 |
| weight food restriction 3 | 0.418 |
| sweating 3 | 0.387 |
| frustration 3 | 0.495 |
| ipo 3 | 0.812 |

| | MR1 |
|---|---|
| SS loadings | 2.243 |
| Proportion Var | 0.374 |

**Legend. Loadings**: standardized factor loadings, correlations between items and the extracted factor. Loadings ≥ 0.40 are typically interpreted as salient. **SS loadings**: sum of squared loadings for each factor. **Proportion Var**: proportion of variance explained. **MR**: **minimum residual (minres)** estimator. **MR1**: Factor 1 from the minres Exploratory Factor Analysis.

**Table S9.** Structural validity and internal consistency for analysis 3 and specific symptoms. Gastrinoma/Non-functioning (PNET.15) internal consistency.

| Raw alpha | Std alpha | Average r | S/N |
|---|---|---|---|
| 0.7243 | 0.7404 | 0.2628 | 2.853 |

| item | n | mean | sd | Raw r | r drop | Alpha if deleted raw | Alpha if deleted std |
|---|---|---|---|---|---|---|---|
| Gut 3 | 66 | 17.42 | 15.52 | 0.6706 | 0.5518 | 0.6743 | 0.6884 |
| Muscle energy 3 | 66 | 23.23 | 22.24 | 0.7503 | 0.5975 | 0.6521 | 0.6802 |
| Weight food restriction 3 | 66 | 8.838 | 13.47 | 0.5058 | 0.3758 | 0.7064 | 0.7248 |
| Sweating 3 | 66 | 8.586 | 17.84 | 0.4628 | 0.2776 | 0.7234 | 0.7436 |
| Frustration 3 | 66 | 14.14 | 20.31 | 0.6681 | 0.5028 | 0.6772 | 0.7009 |
| Gas NF 3 | 66 | 7.912 | 12.44 | 0.6498 | 0.5539 | 0.6831 | 0.6915 |
| Itching 3 | 66 | 10.1 | 17.51 | 0.4267 | 0.2402 | 0.7298 | 0.746 |
| Nocturia 3 | 66 | 18.69 | 24.9 | 0.604 | 0.3681 | 0.7173 | 0.726 |

**Legend. Raw alpha:** scale-dependent α. **Std alpha:** standardized α, unit-free. **Average r:** average inter-item correlation. **S/N**: signal-to-noise ratio (α/(1–α)). **N:** sample size (**n**). **SD**: Standard Deviation. **Raw r**: item–total correlation. **r drop:** corrected item–total correlation, excluding the item itself. **Alpha if deleted**: α if that item were removed; raw/standardized.

**Table S10.** Structural validity and internal consistency for analysis 3 and specific symptoms. Parallel exploratory factor analysis for Gastrinoma/Non-functioning (PNET.15)

Parallel analysis suggests that the number of factors = 1 and the number of components = NA
Parallel analysis indicated a 1-factor solution (observed eigenvalues exceeded random-data eigenvalues only for the first factor).

Loadings:

| | MR1 |
|---|---|
| gut 3 | 0.656 |
| muscle energy 3 | 0.754 |
| weight food restriction 3 | 0.398 |
| sweating 3 | 0.317 |
| frustration 3 | 0.630 |
| Gas NF 3 | 0.664 |
| itching 3 | |
| nocturia 3 | 0.415 |

| | MR1 |
|---|---|
| SS loadings | 2.347 |
| Proportion Var | 0.293 |

**Legend. Loadings**: standardized factor loadings, correlations between items and the extracted factor. Loadings ≥ 0.40 are typically interpreted as salient. **SS loadings**: sum of squared loadings for each factor. **Proportion Var**: proportion of variance explained. **MR**: **minimum residual (minres)** estimator. **MR1**: Factor 1 from the minres Exploratory Factor Analysis.

**Table S11.** Structural validity and internal consistency for analysis 3 and specific symptoms. Analysis of the common symptoms set (internal consistency).

| Raw alpha | Std alpha | Average r | S/N |
|---|---|---|---|
| 0.7064 | 0.75 | 0.3333 | 3 |

| item | Raw r | Std r | r drop | r cor | mean | sd | Alpha if deleted raw | Alpha if deleted std |
|---|---|---|---|---|---|---|---|---|
| Gut 3 | 0.7107 | 0.7173 | 0.5418 | 0.6346 | 16.25 | 16.47 | 0.6341 | 0.695 |
| Muscle energy 3 | 0.8085 | 0.7833 | 0.6293 | 0.7635 | 19.36 | 21.26 | 0.5936 | 0.6677 |
| Weight food restriction 3 | 0.5003 | 0.5697 | 0.3371 | 0.4192 | 7.239 | 12.18 | 0.6956 | 0.7476 |
| Sweating 3 | 0.5444 | 0.488 | 0.2627 | 0.3063 | 9.428 | 20.23 | 0.7349 | 0.7727 |
| Frustration 3 | 0.7174 | 0.685 | 0.5162 | 0.6147 | 11.11 | 19.05 | 0.6402 | 0.7074 |
| Specific symptoms 3 | 0.67 | 0.7565 | 0.608 | 0.7064 | 5.338 | 6.501 | 0.6766 | 0.6791 |

**Legend. Raw alpha:** scale-dependent α. **Std alpha:** standardized α, unit-free. **Average r:** average inter-item correlation. **S/N**: signal-to-noise ratio (α/(1–α)). **N:** sample size (**n**). **SD**: Standard Deviation. **Raw r**: item–total correlation. **r drop:** corrected item–total correlation, excluding the item itself. **Alpha if deleted**: α if that item were removed; raw/standardized.

**Table S12.** Structural validity and internal consistency for analysis 3 and specific symptoms. Analysis of the common symptoms set (parallel factor analysis).

Parallel analysis suggests that the number of factors = 1 and the number of components = NA
Parallel analysis indicated a 1-factor solution (observed eigenvalues exceeded random-data eigenvalues only for the first factor).

Loadings:

| | MR1 |
|---|---|
| gut 3 | 0.621 |
| muscle energy 3 | 0.796 |
| weight food restriction 3 | 0.424 |
| sweating 3 | |
| frustration 3 | 0.630 |
| specific symptoms 3 | 0.725 |

| | MR1 |
|---|---|
| SS loadings | 2.212 |
| Proportion Var | 0.369 |

**Legend. Loadings**: standardized factor loadings, correlations between items and the extracted factor. Loadings ≥ 0.40 are typically interpreted as salient. **SS loadings**: sum of squared loadings for each factor. **Proportion Var**: proportion of variance explained. **MR**: **minimum residual (minres)** estimator. **MR1**: Factor 1 from the minres Exploratory Factor Analysis.
